# Towards more transparent risk assessment of communicable diseases – Redefining probability and impact

**DOI:** 10.1017/S0950268825000147

**Published:** 2025-02-12

**Authors:** Maarten Nauta, Lasse Engbo Christiansen, Stine Kjær Lefèvre, Charlotte Louise Munkstrup, Johanna Young, Hanne Rosenquist

**Affiliations:** 1 Epidemiological Infectious Disease Preparedness, Statens Serum Institut, Copenhagen S, Denmark; 2 ECDC Fellowship Programme, Field Epidemiology path (EPIET), European Centre for Disease Prevention and Control (ECDC), Stockholm, Sweden

**Keywords:** Risk assessment, Rapid risk assessment, Uncertainty, Public health, Emerging infections, Infectious disease control

## Abstract

Epidemic preparedness requires clear procedures and guidelines when a rapid risk assessment of a communicable disease threat is requested. In an evaluation of past risk assessments, we found that modifications to existing guidelines, such as the European Centre for Disease Prevention and Control’s (ECDC) rapid risk assessment operational tool, can strengthen this process. Therefore, we present alternative guidelines, in which we propose a unifying risk assessment terminology, describe how the risk question should be phrased by the risk manager, and redefine the probability and impact dimension of risk, including a methodology to express uncertainty. In our approach, probability refers to the probability of the introduction of a disease into a specified population in a specified time period, and impact combines the magnitude of spread and the severity of the health outcomes. Based on the collected evidence, both the probability of introduction and the magnitude of spread are quantitatively expressed by expert judgements, providing unambiguous risk assessment. We advise not to summarize the risk by a single qualification as ‘low’ or ‘high’. These alternative guidelines, which are illustrated by a hypothetical example on mpox, have been implemented at Statens Serum Institut in Denmark and can benefit other public health institutes.

## Introduction

Within public health, risk assessment (RA) plays a vital role in adequately informing decision-makers on the current scientific knowledge related to public health threats. Requests for RA can require immediate answers in case of emerging threats or incidences, specifically in case of potential communicable disease epidemics. For that reason, the European Centre for Disease Prevention and Control (ECDC) developed its operational tool on rapid risk assessment (RRA) methodology [1], targeted at both national public health experts and experts responsible for rapid assessment of communicable disease threats at the European level. These guidelines, built on general principles of RA [2,3], aim to facilitate the structured and reproducible development of RRAs for communicable disease incidents. The proposed RRA methodology consists of five stages: Define the risk questions; Collect and validate event information; Literature search and extraction of evidence; Appraise evidence; and Estimate risk. For the last stage, decision trees are provided to qualitatively characterize the risk in two dimensions, probability and impact, which are later combined in a risk-ranking matrix to obtain a risk estimate.

At Statens Serum Institut (SSI), which is responsible for the Danish preparedness against infectious diseases in humans, the RA methodologies used until recently were usually chosen on a pragmatic and ad hoc basis. Although there was a strong emphasis at SSI to follow the ECDC RRA methodology due to their operational similarities (i.e. addressing potential public health concerns in a timely manner), various challenges arose when applying these methods. We realized that improved guidelines could harmonize our RAs, increase transparency, and thereby facilitate decision-making. We studied the use of ECDC’s operational tool, as well as guidelines, tools, and manuals published by other international public health organizations [1,2,4–7]. For two typical RAs that had previously been performed at SSI, one on seasonal influenza [8] and one on mpox [9], case studies on the implementation of the ECDC operational tool were performed, to evaluate how this would impact the RA, while comparing ECDC’s RRA methodology with alternatives suggested elsewhere.

From that experience, we concluded that an alternative approach could provide more transparent and more informative estimates for decision-making. First, we realized the importance of a clear and unambiguous risk question, which is a prerequisite for understanding the risk estimates obtained. Second, we found a particular challenge in the ambiguity of the definitions of the two dimensions of risk: probability and consequence. This ambiguity emerges from the fact that, instead of two dimensions, RA in infectious disease epidemiology often considers three: the probability of introduction, the magnitude of spread, and the severity of the consequences. These three dimensions are not explicitly recognized in the ECDC RRA methodology. In line with this challenge, it was unclear whether ‘probabilities’ in the ECDC guidelines [1] referred to populations or individuals, with the potential to mix up the probability of introduction with the probability and magnitude of the spread of an infectious disease. Hence, it appeared that the two dimensions of risk, probability and impact, could be interpreted in different ways, depending on the context of the RA and the involved expert’s background, leading to a lack of clarity on the interpretation of the decision trees provided [1]. Third, the questions in the decision trees include subjective terminology, such as ‘likely’ and ‘significant’, which may induce inconsistency in the assessment due to different interpretations of the words. Last, by expressing the probability and impact in qualitative terms, and combining these in a single risk estimate, the RA may become less transparent and implicitly enter the risk management domain.

In this article, we summarize and discuss our alternative guidelines and focus on the modifications to ECDC’s RRA methodology, which aim to increase the transparency of the process and enhance the quality of the RA for the involved risk assessors and stakeholders, by providing clear definitions and using quantitative expressions where possible. For illustration, we show an example based on an RA on the introduction and spread of mpox in Denmark, using these alternative guidelines.

## Methods: Alternative risk assessment guidelines

### Unifying risk assessment terminology

A crucial aspect of RA is its place in the risk analysis framework, where RA is the responsibility of independent experts which provide scientific advice to decision-makers, the risk managers. The risk assessor’s role implies that the RA evaluates risks and potential risk mitigation strategies solely based on the available evidence, without otherwise influencing the decision-making process. A comparison of guidelines, tools, and manuals from different public health organizations quickly showed that terms and definitions within risk analysis can be different within different areas of expertise [3,10,11]. This can easily be a source of misunderstanding and requires that the terminology is well-defined. Definitions used here are therefore given in Table 1.

**Table 1.** Definitions of terms used in risk analysis. They were selected from definitions used by different organizations, as those that are most suitable for our methodology. The last four are specific for this methodology

Hazard	An agent that has potential to cause adverse health effects in exposed populations [2]
Probability	Defined depending on philosophical perspective: (1) the frequency with which sampled values arise within a specified range or for a specified category; (2) quantification of judgement regarding the likelihood of a particular range or category. [12]
Risk	The likelihood of the occurrence and the likely magnitude of the consequences of an adverse event during a specified period. [2]
Risk analysis	A process consisting of three interconnected components: risk assessment, risk management and risk communication. [12]
Risk assessment	The systematic process of gathering, assessing and documenting information to estimate the level of risk and associated uncertainty related to an event, during a specified period of time and in a specified location. [7]
Rapid risk assessment	Risk assessment with limited time for (among others) collection and appraisal of evidence, which implies larger uncertainties in the estimates and increases the need for clear risk assessment procedures and guidelines. [authors’ definition]
Risk management	The process, distinct from risk assessment, of weighing policy alternatives in consultation with interested parties, considering risk assessment and other legitimate factors, and, if need be, selecting appropriate prevention and control options. [12]
Threat	A potentially damaging event or incident. [1]
Transparent	Characteristics of a process where the rationale, the logic of development, constraints, assumptions, value judgements, decisions, limitations and uncertainties of the expressed determination are fully and systematically stated, documented, and accessible for review. [13]
Uncertainty	A general term referring to all types of limitations in available knowledge that affect the range and probability of possible answers to an assessment question. Available knowledge refers here to the knowledge (evidence, data, etc.) available to assessors at the time the assessment is conducted and within the time and resources agreed for the assessment. Sometimes ‘uncertainty’ is used to refer to a source of uncertainty, and sometimes to its impact on the conclusion of an assessment. [12]
Probability of introduction	Estimated likelihood that a disease is introduced into a defined population group in a defined period of time, expressed as an interval that captures the uncertainty, for example using the proposed scale. If the disease is already present in the population, this probability is 1 (100%). [authors’ definition]
Magnitude of spread	The expected number of people in the population group that will become infected or ill or end up in a disease state categorized in the ‘severity of disease’ scale, given that the disease is introduced into the population group, within a defined period of time. It is expressed as a predefined range; more ranges can be selected if that captures the uncertainty. [authors’ definition]
Consequence (of disease) or health outcome	A selection of categories, related to the pressure on the healthcare system. ‘symptomatically ill’, ‘symptomatically ill seeking health care’, ‘hospitalized’, ‘in intensive care unit’, ‘death’. [authors’ definition]
Impact	Combination of expected magnitude of spread and severity of disease, expressed as ‘very low’, ‘low’, ‘moderate’, ‘high’ or ‘very high’, based on a scoring table. [authors’ definition]

### Steps in risk assessment

**Figure 1.**
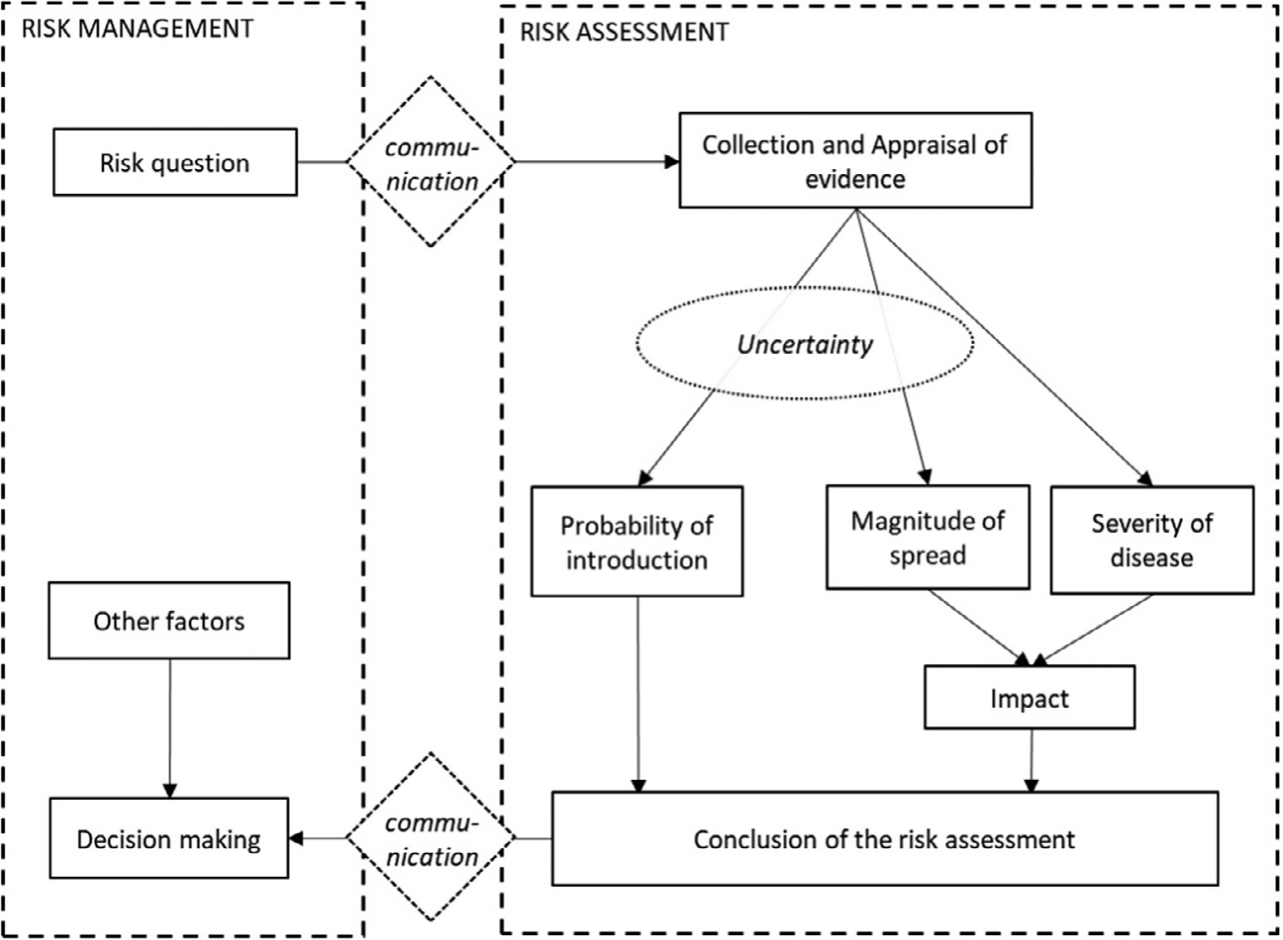
Overview of the risk analysis process. Risk managers and risk assessors have separate roles; whilst risk assessment is independent, communication with risk managers is crucial. The task of the RA is to answer the risk question by collecting and appraising the scientific evidence and assessing the probability of introduction and the impact of the disease and the attending uncertainty.

After identifying a potential communicable disease threat, risk managers typically request a RA, which should be provided within a restricted timeframe, ranging from a few days to a few months. The RA is done based on up-to-date scientific knowledge, after evaluation of the evidence by a group of scientific experts, that cover the relevant areas of expertise. Our alternative guidelines propose to follow the steps outlined in Figure 1. Among these steps, ‘probability of introduction’ and ‘impact’ capture the two dimensions of risk. An important difference with ECDC’s RRA [1] is that our definition of probability of introduction explicitly specifies the population(s) and period of time to be covered by the RA. ‘Impact’ covers both the magnitude of spread in the population and the severity of the disease. We choose to use these definitions to avoid confusion between experts, which we experienced in our case studies, as, depending on the context, the magnitude of spread may both be part of the probability dimension and the impact dimension. As part of the evidence appraisal, the experts consider the uncertainty in attending the probability of introduction and the impact. This uncertainty is expressed by using numerical intervals for the probability of introduction and magnitude of spread within different severity classes, as explained in Sections “Probability of introduction” to “Uncertainty”.

#### Risk question

It is crucial for any RA to clearly define the risk questions. In general, such a question refers to an outcome or quantity that could (in principle) be observed or measured without ambiguity in the real world or obtained from a defined scientific procedure [12]. Here, we refer to the type of risk questions that are most commonly asked to SSI, concerning (re-)emerging communicable disease threats. It is further assumed that the question requires an assessment of a risk, that refers to the probability and impact of an event.

Whereas ECDC’s operational tool [1] only indicates that the RA should be performed separately for all specific population groups and geographical areas, the Joint Risk Assessment Operational Tool [7] provides more detailed guidance for phrasing ‘specific, relevant and time-bound’ risk questions, by including the ‘what’, ‘where’, ‘when’, and ‘how’ of the risk. Here, ‘what’ refers to the hazard (i.e. the pathogen) and the event (e.g. the death of a predefined number of people), ‘where’ refers to the populations(s) and geographical region(s) (e.g. the adult population in Denmark), ‘when’ refers to the timeframe (e.g. the coming year) and ‘how’ refers to the source of the hazard (e.g. a specific animal population). An example of a risk question would be: ‘*What is the probability and impact of at least one person in Denmark being infected by influenza A (H7N9) virus from wild birds within the next 6 months?*’

In line with [7], our guidelines cover all these elements in the risk question(s), as this clearly defines the scope of the RA, allows fit-for-purpose RA, and supports efficient use of the available time and resources. While the final responsibility for the question lies with the risk managers, the risk assessors are often more aware how a well-defined risk question is to be formulated, and can better assess the feasibility of answering it within the available timeframe. Therefore, it is crucial that risk managers and assessors agree on the interpretation of the question in the initial phase of the RA.

#### Collection and appraisal of evidence

A crucial part of the work of the scientific experts involved in the RA is the efficient collection and appraisal of evidence required to answer the risk question(s). For this activity, our guidelines do not prescribe any alternative approach to ECDC’s operational tool [1], where three of the five stages in the RRA methodology provide detailed guidelines.

To answer the risk question(s), the quality and representativeness of the collected evidence should be transparently communicated, as this significantly influences how certain the conclusions are. Public Health England [5] and ECDC [1] provide a useful classification in terms of ‘good’, ‘satisfactory’, or ‘unsatisfactory’ quality of evidence, which is made by the experts based on the collected information. The judgement on the quality of evidence has to be taken along when the uncertainty in the conclusions of the RA is characterized by the experts (see below).

#### Probability of introduction

In the actual RA, the first dimension of the risk is the probability. We address the case when the risk question(s) relate to a human disease that may be (re-)introduced into a population as defined in the risk question, due to a communicable disease threat from outside. To cover the probability dimension of risk, we therefore request an estimate of the probability of introduction in a population group and geographical area, within a defined time period, that is the probability that one or more people in the targeted population will get infected. Its estimate should be based on the collected evidence, which may include data and model predictions, provided by the scientific experts. A suitable method for expert knowledge elicitation may be used [14]; if time is limited, an estimate may be obtained by discussion between the experts.

Probability is defined as a number between 0 and 1, and therefore the only transparent way to communicate it is to use a quantitative expression [12]. As it is challenging to provide a precise numerical point estimate of a probability, we propose to use the probability scale in Table 2, which is derived from the guidance that the European Food Safety Authority (EFSA) uses [14]. The scale includes verbal expressions defined by intervals of probabilities. These numerical intervals are used, because the estimates are generally uncertain, and experts commonly think in approximate terms. Experts can combine intervals in the table when these are considered more appropriate.

**Table 2.** Definitions used for the probability of introduction of a disease in the population(s) and time period defined in the risk question. Introduction is certain if the disease is already known to be present in the population

Qualitative term	Quantitative term (% probability range)
Certain	100
Almost 100% likely	99 to <100
Extremely likely	95–99
Very likely	90–95
Likely	66–90
As likely as not	33–66
Less likely	10–33
Not likely	1–10
Very unlikely	0.1–1
Extremely unlikely	0.001–0.1
Almost impossible	<0.001

#### Impact

The second dimension of the risk is the impact, expressing the public health consequences of the introduction of the disease. It is a combination of two underlying dimensions, the magnitude of spread in the population and the severity of the disease (Figure 1), obtained by expert judgement of the scientific experts involved in the RA. After consulting the collected evidence, these experts assess how many people (or which fraction) in the defined population(s) are expected to get infected and/or end up in different health states within the time period indicated in the risk question, given that the disease is introduced in the population. If available, infectious disease models may be applied to support these assessments. Based on risk questions that we received during epidemics in the past, we define these health states as five different classes of consequences (or health outcomes) with increasing severity: *symptomatic disease, symptomatic seeking health care, hospitalization, admission to the Intensive Care Unit (ICU), and death.* In each assessment, the relevant classes for the particular question are selected. Based on the evidence, which may include data and model predictions, the scientific experts have to estimate how many people from the different population groups are expected to end up in each consequence class. The overall impact of the spread of the disease is derived from the magnitude of spread in the different consequence classes and characterized as ‘very low’, ‘low’, ‘moderate’, ‘high’, or ‘very high’, as defined in Table 3. This characterization is subjective, based on discussion between the authors, using different examples of (potential) outbreaks, including the case studies on influenza and mpox.

**Table 3.** Impact table, used to characterize the impact based on magnitude of spread (incidence rate) and consequence classes (five health outcomes). Impacts are defined by the incidence rate (upper line in the heading), but in practice experts may prefer to use the absolute incidence; in the table we illustrate this for a hypothetical population of 200.000 people (lower line in the heading)

	Magnitude of spread						
Consequence class	0.2^1^-1	1–10	10–100	100–1000	1000–50000	>50000	incidence rate per million
	–	1–2	2–20	20–200	200–10,000	>10000	Incidence per 200.000
Symptomatic	Very low	Very low	Very low	Very low	Low	Moderate	
Seeking healthcare	Very low	Very low	Very low	Low	Moderate	High	
Hospitalization	Very low	Very low	Low	Moderate	High	Very high	
ICU	Very low	Low	Moderate	High	Very high	Very high	
Dead	Low	Moderate	High	Very high	Very high	Very high	

1 The lower limit 0.2 per million is chosen because it reflects 1 person in a population of 5 million, the approximate size of the Danish population.

Note that the magnitude of spread is expressed as an expected incidence rate, that is the affected number of people per million, given in the first row of Table 3. Hence, the characterization of the overall impact is based on the incidence rate, not on the absolute incidence (i.e. the total number of cases in the second row of Table 3). This absolute incidence is just given to facilitate the assessment. Note that intervals for the incidence rates are used as there will be uncertainty associated with these estimates.

The overall impact is evaluated for all consequence classes where at least one case is expected. Hence, we obtain up to five impacts, one for each consequence class. The highest of these is selected as the final overall impact of the disease for the population considered. Risk assessors can therefore focus on the combination of the expected number of affected people and the consequence that are expected to give the highest impact.

#### Uncertainty

RAs are always uncertain. This uncertainty is a consequence of limited knowledge and limited quality of evidence, as well as stochasticity or randomness. The assessors should consider all uncertainties that play a role in the assessment and their impact on the conclusions. One option to facilitate this is to make a table with identified uncertainties and evaluate their effect on the estimate of the probability of introduction and/or the impact.

In the proposed approach, uncertainty is expressed in the intervals used when estimating the probability of introduction, and the intervals of numbers of people with different health outcomes for the magnitude of spread. As long as uncertainty is captured by these intervals, single outcomes can be obtained in the impact scale. If uncertainties are larger, the assessors can decide to characterize the impact by intervals as well, with the option to explicitly indicate the most likely one. For example, the impact can be expressed as ‘moderate to high, most likely moderate’, if the impact table (Table 3) indicates that that would be the case.

#### Conclusions

The RA conclusions should be short, and directly answer the risk question(s). A table can be presented that provides the estimates for the probability of introduction and the impact for, for example, different (combinations of) populations or strain types, and other relevant information can be added. Additionally, the outcomes are described and put into context. It will often be useful to pick out important examples from the table and explain the indicated results in terms of magnitude of spread and consequences, using quantitative expressions if possible. Additional perspectives may be added, but it should be critically evaluated to what extent their inclusion is relevant and falls within the responsibility of the RA.

## Results

### Mpox example

For illustration, we provide an example of an adapted version of the RA on the introduction and spread of mpox in Denmark for explanatory purposes. This example is based on an RA performed at SSI in August 2022 [9], before the alternative approach was developed. At that time, mpox clade 2B was spreading in Europe, and the Danish health authorities requested an RA from SSI. In this case study, we redid this RA, first to evaluate ECDC’s operational tool and later to pilot our proposed methodology. Here we report on the latter exercise. Note that this is to be considered a hypothetical RA, as the focus was on the method, and it was not performed by the team of disease experts involved in the original RA.

#### Risk question(s)

A suitable question for the RA would be:

*What is the probability of introduction of an mpox infection into the following population groups in Denmark in the coming two months, a) men who have sex with men (MSM) with many sexual contacts, b) other groups with many sexual contacts, c) health care professionals, d) pregnant women and immunocompromised persons, e) children, and f) other population groups.*

*Given that mpox is introduced in a population group, what is the public health impact for this population group in the following two months?*

Note that the question refers to the *hazard* (mpox) and the *event* (introduction of the infection and its impact), the specific *populations* in Denmark, and a *time* frame. All *sources* of mpox infection are to be considered; for populations a) and b), the route of transmission is implicit.

#### Collection and appraisal of evidence

In the summer of 2022, a detailed overview of the current situation of the mpox epidemic could be given based on national and international surveillance data, and disease characteristics based on peer-reviewed literature, submitted research papers, and reports of recognized authoritative institutes, such as ECDC. Therefore, the quality of evidence can be regarded as ‘good’.

#### Probability of introduction

The probability refers to the introduction into each of the six predefined population groups, that is the probability that at least one person in the population group in Denmark will be infected by mpox. This probability of introduction varies widely from certain (100%, in MSM with many sexual contacts, where the disease was already known to be present) to extremely unlikely (0.001–0.1%) in population groups where the type of contact required for transmission is not expected.

#### Impact

The impact estimate combines the magnitude of spread in each specific population, given that the disease is introduced in this population and the health outcomes of the disease.

The mpox virus is predominantly transmitted by close physical contact. The magnitude of spread is therefore assessed to be largest within the population groups MSM and others with many sexual contacts, whereas infection in remaining population groups will mainly be ‘spill-over’-events.

For each population group, the expected number of infected people that provides a specific burden on the healthcare system is assessed by the experts. We illustrate this assessment for two examples, the MSM groups and healthcare personnel (Table 4).

**Table 4.** Impact table for the population groups ‘MSM with many sexual contacts’ (A) and ‘health care personnel’ (B). The incidence rate (per million) is translated into an incidence per estimated population group size (i.e. 5000 (A) and 100000 (B)), which is used by the experts to facilitate their assessment. The assessed impact per consequence class is given in bold italics. No cases are expected in ‘ICU’ and ‘dead’

A							
	**Magnitude of spread**						
Consequence class	0.2–1	1–10	10–100	100–1000	1000–50000	>50000	incidence rate per million
	–	–		1–5	5–250	>250	incidence per 5000
Symptomatic	Very low	Very low	Very low	Very low	***Low***	Moderate	
Seeking healthcare	Very low	Very low	Very low	Low	***Moderate***	High	
Hospitalization	Very low	Very low	Low	***Moderate***	High	Very high	
ICU	Very low	Low	Moderate	High	Very high	Very high	
Dead	Low	Moderate	High	Very high	Very high	Very high	

B							
	**Magnitude of spread**						
Consequence class	0.2–1	1–10	10–100	100–1000	1000–50000	>50000	Incidence rate per million
	–	–	1–10	10–100	100–5000	>5000	Incidence per 100000
Symptomatic	Very low	Very low	***Very low***	***Very low***	Low	Moderate	
Seeking healthcare	Very low	Very low	***Very low***	***Low***	Moderate	High	
Hospitalization	Very low	***Very low***	***Low***	Moderate	High	Very high	
ICU	Very low	Low	Moderate	High	Very high	Very high	
Dead	Low	Moderate	High	Very high	Very high	Very high	

*MSM with many sexual contacts*: Based on Danish population data, it is estimated that this group consists of 5000 people. As indicated in Table 4A, based on the collected evidence, the scientific experts involved in the RA assess that 5–250 people of 5000 in this group will be symptomatically ill and seek healthcare, which, according to Table 3, implies ‘low’ and ‘moderate’ impacts. Of those, 1–5 are assessed to require hospitalization (‘moderate’ impact), whereas none are expected to require ICU or die. This means that the overall impact is scored as ‘moderate’, the highest of the scored impacts.

*Health care professionals*: Based on Danish population data, it is estimated that this group consists of 100000 people. In this case, the uncertainty about the number of people that will end up in the different consequence classes is large, so the experts can use wider ranges of impact than the predefined ones. This is illustrated in Table 4B. Here, between 1 and 100 people are expected to be symptomatic or seek health care, and between 0 and 10 are expected to be hospitalized. Following this assessment, the overall impact for health care professionals, given that the disease is introduced in this population group, would be ‘very low to low’, the highest of the scored impacts.

Examples for the estimates for all population groups, obtained in a similar way, are given in Table 5.

**Table 5.** Estimates for the probability of introduction and impact for the six population groups

Population groups	MSM with many sexual contacts	Other with many sexual contacts	Health care professionals	Pregnant women and immuno-compromised	Children	Other population groups
Probability of introduction	Certain (100%)	Likely (66–90%)	Very unlikely (0.1–1%)	Extremely unlikely (0.001–0.1%)	Extremely unlikely (0.001–0.1%)	Extremely unlikely (0.001–0.1%)
Impact	Moderate	Moderate	Very low–low	Low	Very low–low	Very low

#### Conclusions

The conclusions could for example be formulated as:

‘*SSI assessed the probability of introduction of mpox in Denmark and the public health impact after introduction, for different population groups. The quality of the evidence considered for the assessment is graded as ‘good’.*

*Among MSM, mpox has been found since 22 May 2022. Based on knowledge on the transmission routes, mpox is expected to spread within this group, with a moderate public health impact. It is assessed that between 5 and 250 persons will be symptomatically ill and seek healthcare, and between 1 and 5 will be hospitalized in the coming two months.*

*In other population groups, mpox has not yet been detected. SSI assesses that mpox is likely (66–90% probability) to spread to others with many sexual contacts, but very unlikely (0.1–1%) to spread to healthcare workers and extremely unlikely (0.001–0.1%) to spread to other population groups in Denmark. If introduced in these population groups, based on the available evidence, the public health impact is assessed to be moderate for others with many sexual contacts (between 10 and 500 symptomatically ill) and very low for the rest of the Danish population (between 1 and 50 persons symptomatically ill and seeking healthcare)*’.

Note that these conclusions summarize the estimates for the probability of introduction and the impact separately without reference to an overall risk. The most notable quantitative estimates are given to clarify the verbal expressions such as a ‘very unlikely’ probability of introduction and a ‘moderate’ impact for the population group ‘others with many sexual contacts’. As the impact categorization is based on the incidence rate, and not on the incidence (i.e. on the relative number of cases and not on the absolute numbers), the numbers associated to the different impact categories may be different between population groups.

Relevant context can be added to these conclusions, if the experts consider this appropriate, for example in relation to preventive measures, long-term developments, and so on.

## Discussion

In this article, we summarize an alternative approach to ECDC’s RRA that has been introduced at SSI in Denmark. It was proposed after we experienced challenges implementing the ECDC operational tool [1] and aims to offer specific definitions and procedures that should facilitate the process, increase the transparency of the RA and support the subsequent risk management process. It uses elements of risk assessments used in other areas that extensively apply RA, such as food safety and animal health.

In our approach, the two dimensions of risk are explicitly defined as the *probability* of introduction in a specified population, in a specified period of time, and *impact*, which captures both the magnitude of spread in the population (expressed as incidence rate) and the severity of the disease (defined in five consequence classes). These definitions should prevent confusion on the probability and consequences referred to in the RA. In case of an existing threat that increases within a population where it is already known to be present, our approach suggests to consider this increase as part of the magnitude of spread and thus as part of the impact, instead of the probability dimension of the risk. This may not always be intuitive, but it ensures consistency in the RA methodology. Our approach allows quantitative expressions of the estimates, which increase the transparency of the assessment. These quantitative estimates are ideally derived from quantitative data, but if these are not available, they can be based on expert judgement as well, a method extensively used by EFSA [12,14]. Although it may be challenging for scientific experts to provide such quantitative estimates by expert judgement, the use of ranges assures that very precise estimates are not needed, and uncertainty can be acknowledged. It is beneficial to add a facilitator to the team of experts, who is familiar to the RA process, can give guidance in providing quantitative assessments, and guards the process of expert knowledge elicitation [14].

When characterizing the impact, we propose to assess the magnitude of spread on the basis of incidence rate, and not on the absolute incidence. This implies that, for example, in a subpopulation of 200000, 2–20 deaths will result in a ‘high’ impact score, where in a subpopulation of 2000000 the same number deaths only scores ‘moderate’. This difference may be interpreted as if people in the first subpopulation are valued higher than those in the second. However, this approach ensures that individuals in all population groups are treated equally. The alternative would be that the same risk gets less weight in smaller (minority) populations, which can be interpreted as discriminatory. It is therefore proposed to explicitly refer to the quantitative estimates associated with the highest impacts, as in the mpox example, to ensure that the risk manager is aware of the numbers behind the assessed impact.

Another element of the impact is the severity of the health outcomes. Here we defined categories that were deemed to be suitable for Denmark. In these definitions, critical parameters as the national or regional health systems’ hospital or ICU capacity are not explicitly included, as these are likely to be variable and not readily available. Using our approach, in specific cases and outbreak situations, the impact may quite easily be evaluated against these parameters and communicated to the relevant risk managers.

Risk assessors should be transparent about the uncertainty when the conclusions are formulated, as a good characterization of the uncertainty is of crucial importance for risk managers. Such characterization ideally implies a quantitative approach [12]. It is inappropriate to only use a verbal expression such as ‘…however, the uncertainty is large’, as this only reads as a disclaimer, is highly ambiguous, and shifts the responsibility of interpreting the uncertainties described in the RA to the risk managers. Therefore, our methodology explicitly uses numerical intervals to express the probability of introduction and the magnitude of spread in different consequence classes, even in the absence of quantitative estimates from data, statistical analyses or models.

Purposely, there is no proposal for a combined risk matrix or other method to conclude the RA by a single risk estimate which characterizes the risk as ‘high’, ‘low’, or otherwise. Although such an approach is proposed elsewhere [1,4,6,7] and it may be useful in the context of risk ranking, we believe it has little added value. Moreover, a disadvantage of such an approach would be that it reduces a multidimensional outcome of an assessment into one single dimension, which obscures important information for the risk manager. Additionally, words indicating the level of risk are subjective and may guide the risk managers in their decision. In general, if a RA concludes that a risk is ‘low’, it suggests that risk mitigation is of minor relevance, whereas a ‘high’ risk cannot be ignored. By using such terminology, the risk assessors may inappropriately enter the risk management arena.

The proposed methodology is now being applied at SSI in Denmark and will regularly be evaluated. Obviously, it is only aimed at risk questions in the area of (re-)emerging communicable public health threats that are in line with the methodology. Therefore, our approach is particularly useful when specific populations within a geographical region are addressed, as in RAs performed by national public health institutes. We foresee that flexibility in definitions and alternative approaches may be required when the scope is extended to, for example zoonotic or endemic diseases, which would be a welcome development. Meanwhile, our revised methodology can facilitate other public health institutes in performing more transparent RA and support preparedness activities across the world.

## Data Availability

The presented results are obtained through discussion on the referenced evidence. No additional data has to be made available.
